# Abstracts and Gleanings

**Published:** 1888-09

**Authors:** 


### ABSTRACTS AND GLEANINGS.

**Change of Life and its Neurosis.**—Prof. William Goodell, A. M., M. D., *in Polyclinic.* The menopause occurred ten years ago. She complains of a burning pain in the pelvis. On vaginal examination, I find a cicatricial band at the neck of the womb. We not infrequently find women about this age complaining of a burning pain in the abdomen, running down through one iliac region of the vulva. This, to my mind, is a neurosis, and it is very difficult to cure. The change of life usually does not require a long time, and, as a rule, at the end of that time the woman is well; but she may present a condition of this kind. Under these circumstances I always give the bromides, and a favorite prescription with us is the following:

℞. Ammonii bromidi..............................ʒ iv,

Ammonii chloridi.............................ʒ ij,

Tr. gentianæ..................................

Aquæ.............. ..........................aa℥ iij.

M. Sig—A teaspoonful, in water, before each meal.

I always give with the bromides a bitter tonic, to counteract their depressing effect. I am fond of using the ammonium chloride, on account of its stimulating effect on all the emunctories. Another formula, which I frequently employ in these cases, I may as well give you now. It is my pil. sumbul. comp:

℞. Acidi arseniosi..............................gr. j,
Ferri sulph exsic............................
Ext. sumbuli..................................aa gr. x4,
Assafœtidæ.....................................ʒ iv.

Ft. pil. No. 11. M. Sig.—One after each meal. If this does not have the desired effect, the dose may be increased.

I am disposed to think that the burning of which this patient complains is purely neurosis. It seems incredible that at this time of life it could come from the ovaries, but it may come from the plexus of nerves in the neighborhood of these organs There is another form of burning to which I desire to refer. Women about the change of life, or past it, will speak of a burning of the vulva, usually accompanied by itching.

My advice is, under such circumstances, always to examine the urine for sugar. If the woman is at all stout, there is probably sugar in the urine. It has been supposed that it was the presence of the sugar in the urine trickling over the parts that caused the pruritus. This may be so in a few cases, but in the majority of instances the itching is a neurosis. In the treatment of these cases, local applications, with remedies directed to the glycosuria, is required.

There may be at this period of a life a burning, accompanied with itching, which may be due to a senile catarrh, with an acrid leucorrhœ. The discharge comes from the cavity of the womb, and while it may not be sufficient to attract attention, it may be sufficient to cause itching. I have found that curetting the womb was the best way of getting rid of this form of burning. With this I associate internal applications.

These women will often come to you with the statement that they have a tumor, when the whole trouble is that they have nervous flatus, causing disten ion of the abdomen. I wish that I could find a theory which would satisfactorily explain how it is that, in cetain conditions of the nervous system, there will be the sudden distention of the abdomen with flatus. I am disposed to think that gas may be rapidly generated in the human body. Otherwise it seems impossible to explain this. The patient's at-attention is called to the swelling bv the fact that the clohting is tight, and she will come to you with the statement that she has a tumor, when the whole trouble is due to a collection of wind. In such a case there would be a resonance all over the abdomen. This is a diagnostic sign. Another is, that by taking hold of the abdominal walls, you can lift up a large fold of skin, so that there would not be room behind it for a tumor of any size. Then if you percuss, you will find no evidence of a tumor. Of course, this does not serve to exclude a small tumor, but the patient consults you on account of a large swelling.

**Experience with Antifebrin.**—Not seeing much written, and seeing some inquiry about antifebrin, I have concluded to give the readers of the *Reporter* my experience, says Dr. S. R. Humston, in the *Medical and Surgical Reporter*.

CASE I.—Wm. D., age 18, an epileptic, was just getting over an attack which left him with a terrible headache. I gave him four 15-grain capsules, one to be taken every three or four hours until he was easy. He took one at noon, and one at 4 p. m. His head was easy at 6 p. m., and he slept well. The next morning he had a slight headache, and took one capsule. He reported himself as cured that evening.

CASE II.—Mr. V. D., age 57; first stage of typhoid fever with temp. 104°, pulse 80, and severe frontal headache. It was my first visit, and he lived near Wm. D. I had not been in the house long when he asked me if I had any of those capsules for headache that I gave to Wm. D. I said I had, and gave him a 10 gr. capsule, which eased his head, made him sweat terribly, reduced his fever 2°, and brought his pulse down to 56. I thought it was the antifebrin which reduced his pulse, but I found it was not, by leaving it off. I continued to give him 10-grain capsules every afternoon; gave one every three hours, and only one day did he have to take the third dose. He made a good recovery.

CASE III.—Mary B., age 19, has tabes mesenterica, with headache, dry skin, quick pulse, etc. I give her 8 to 10 grains of antifebrin nearly every afternoon, and never had to give the third dose.

I find in antifebrin a medicine which will relieve nearly every kind of headache, and reduce the temperature when there is fever. In the majority of the persons to whom I have given it, it has produced sleep more natural than that caused by opium or chloral.

**Cascara Sagrada in Rheumatism.**—Dr. H. T. Goodwin, M. D., Assistant Surgeon, United States Marine Hospital Service, in the *New York Medical Journal*, June 9th, 1888, says: The effect of cascara sagrada in rheumatism I discovered by accident. About three months ago I was attacked with severe rheumatic pains in my shoulder, the slightest motion causing intense pain. The third day of the attack I commenced taking as a laxative 10 drops of the cascara, t. i. d. The first morning after taking it the pains were so much less severe that I could move my arm freely. The day following I was entirely free of all discomfort.

Although, as I have intimated, I had not taken the cascara with any idea of relieving the rheumatism, it occurred to me a few days later that possibly the sudden subsidence of the pain might have been due to the drug. There being a few cases of rheumatism in the wards, I determined to try to verify my suspicions. Discontinuing the salicylates, iodides, etc., which these patients were taking, I substituted ext. cascaræ sagradæ fl.. 1 c.c., t i.d.(15 gtt.) The patient was a Swedish sailor who had been admitted three months previously. He suffered intensely, and, although almost everything had been given from which relief might be expected, his suffering was not allayed. For a day or two after admission he improved on large doses o' salicylate of sodium, but subsequently the pains returned as badly as ever, and the salicylate had no further beneficial effect. Iodide of potassium was given several different times, but owing to an idiosyncrasy, could be continued only two days at a time, a profuse rash making its appearance over the patient's entire body, the pains remaining as acute as ever. They were not confined to any two or three joints, but felt in all, being more severe, however, in the wrists, finger joints, and ankles, all of which sometimes became œdematous. On the evening of Feb. 5th, I commenced the exhibition of 15-drop doses of cascara sagrada three times daily. The following morning he was about the same; the second day he was much better; on the seventh he was so far recovered that he asked and obtained permission to walk out. From this on he continued to improve steadily, and on the 17th of February was discharged recovered.

I have since used the cascara in fully thirty cases, some ten of which were in out-patients, and, with the exception of three or four in which there was a syphilitic taint, I have obtained the most satisfactory results. I commenced with 1 c. c., t. i. d., and have so far never had to increase it beyond 1.5 c. c., and even to this extent in but two cases. I have seldom had to wait beyond twenty-four hours for beneficial effects. In two cases I had to stop it temporarily owing to its opening the bowels too freely. In such cases I would suggest that one of the preparations of iron be given (separately) at the same time. I usually combine it with syrup or glycerin in equal parts, and instruct the patient to take from 30 to 40 drops in water. In one case in which neither it nor the salicylate of sodium appeared to give much benefit I combined the two with good effect. It is but seldom the bowels are opened too freely by it, the cases above referred to being the only ones I have so far observed.

Among the out-patients upon whom I have used it were two intelligent officers of vessels. One was an old river pilot who had periodically suffered intensely for years. I gave him equal parts of the cascara and syrup. of which I instructed him to take 2 c. c., t. i. d., and requested him to see me again in three days. He returned a month later, and then only to get the medicine renewed. He reported that he had never before had anything relieve him so quickly. The pains began to abate within twenty-four hours after taking the first dose, and in three days after left him entirely. He had had no return, but, for fear of another attack, had come to ask for a bottle to keep with him.

The second case was that of Mr. R., first clerk on a large river steamer. He was suffering so much with pain in the hip-joint and thigh that he could scarcely get to the office. I put him on large doses of salicylate of sodium, with colchicum and iodide of potassium, and instructed him to return in a day or two. In a week he sent a friend to say that the pain, instead of lessening, was so severe that he could not get to the office. The salicylate, etc., were stopped and he was given cascara syrup, 35 drops, t. i. d. This was on Friday afternoon. On Sunday he came to the hospital and reported that he had commenced taking the second prescription Saturday morning, and that on Sunday he had felt decidedly better. He was ordered to continue the drops, and report on Wednesday. Tuesday he sent word that he should be unable to report, as he was sufficiently recovered to resume his usual place on the steamer.

I am not able to explain the action of the drug in relieving rheumatism; I leave that to other observers. I write this in the hope of inducing other medical men to use the cascara, report their experience, and indicate, more particularly, in what class of cases they have found it of most benefit.

**Summer Complaint.**—Dr. Jacobi read a paper before the New York Academy of Medicine on this subject. He regarded summer complaint as a clinical entity, so to speak, and not a pathological entity. He made that point because, he said, it had become the custom to shape our ideas regarding the pathology of this disease, and indeed of disease generally, as to assume that there was but one cause for a large number of maladies. But this, from the clinical stand-point, he thought, was decidedly wrong. The very fact that a large number of bacteria had been discovered, and that a few diseases had been traced to a single bacterium, had led to the assumption that nearly all infectious diseases, and some others, are due to such a cause. This assumption he regarded as probably true in many cases, and it was desirable that it should be so; but he thought physicians had drifted too far with that current. We were inclined, he said, to believe that such diseases as diphtheria, scarlet fever, and so on, are due to invasion of the system by certain bacteria. He said "we are inclined to believe it," but it has never yet been proved; yet it was possible, it was probable, it was even desirable. It would facilitate the study of pathology a great deal, if it were proved. But imagine, he said, one asserting that there could be no imflammatory disease of the respiratory organs without a pneumococcus; that pneumonia under all circumstances, meant a coccus disease. This would certainly be a mistake. The same, he thought, is true of summer complaint. Different forms of inflammation and ulcerations in the intestinal tract give rise to the same clinical symptoms. While it is true there is a form of intestinal inflammation which appears to be due to the formation of bacteria, yet, when speaking of summer complaint, physicians often have in mind a number of diseases which have the same symptoms which do not belong to one and the same anatomical lesion.

A question which is constantly recurring is: whether or not the heat by itself is a sufficient cause of diarrhœa. This direct effect of heat had been denied before the Academy only a short time ago by Dr. Seibert, and the questions of fermentation and bacteria had come to monopolize attention to such an extent as to exclude from vision any other etiological factor.

Dr. Jacobi holds that diarrhœa may be produced by the influence of heat alone, and thinks not unfrequently such cases are among the most severe. Diarrhœa sometimes occurs during sunstroke in the adult. Why solar heat should act on the brain alone, and not on the other parts of the nervous system, he regaded as inexplicable. Infants brought up on good mother's milk should never, according to the bacterial theory, have diarrhœa, yet we know that heat affects them in the following ways: it produces convulsions; it produces convulsions and diarrhœa; it produces diarrhœa. At any age, diarrhœa from neurotic causes is frequent enough. It can be produced by dividing the mesenteric nerve. The center for this paralytic diarrhœa is, he thinks, in the medulla oblongata.—*Medical and Surgical Reporter.*

**Creasote in Phthisis.**—This well-known germicde has been demonstrated by Sternberg to be fatal to micrococci in the strength of 1 to 200. While Sommerbrodt admits that it may not be from its antiseptic or germicidal powers that it benefits, but that it may be simply from its favorable action upon digestion, still he advises that it be pushed to its utmost limit of tolerat on, as here is where so many fail in its use. Pushing it to this extent would be unnecessary if its beneficial action was expended only upon the process of digestion and assimilation; for experience with the drug plainly shows that it is the small doses and not the large which assist digestion, and that the large ones which he advises occas onally irritate the stomach. He says that disappointment arises only through timidity. "The more creasote that can be borne the better the effect," is his dictum. He claims to have treated five hundred cases during the past nine years, and of those treated by bouchard's formula twenty-seven per cent. recovered. Others were treated by the following formula, which gave the best results:

℞. Creasoti.................................*m* xv,

Tr. gentian.................................*m* xlv,

Spir. vini rect..............................fl. ʒ viss,

Vini Xerici q. s. ad.........................fl. ℥ iij.

Of this 1 ounce was taken three times a day. The creasote was gradually increased to 30 grains. He obtained the best results by following treatment for from three months to a year. The most benefit was seen in the young and in the first stages of the disease, when the symptoms were not well defined. Good results were always secured when scrofulous glands were present. It generally relieved irritation and cough, and secretion and expectoration diminished, so that narcotics could be dispensed with.

Dr. J. Solis Cohen has used creasote for many years, not as a specific but to prevent retardation of the decomposition of undigested nutriment. He has used it in chronic diarrhœa for twenty-five years. He has found that the beechwood creasote is the best to use, and rarely exceeds one-half minim at a dose. He claims to have used this with such benefit for twelve years that he rarely uses oil or hypophosphites.

Mrs. G. M., thirty-six years old, was seen first for her present trouble December 3, 1887. I had treated her four years before for incipient phthisis, but had heard nothing from her during the intervening time. On November 1 (one month before I saw her) she gave birth to a child, and since her confinement had rapidly failed until she sank into a condition of tuberculosis in the third stage. Her condition was as follows: Temperature 103° F.; profuse sweating at times; violent cough and abundant expectoration of yellow muco-pus; violent delirium occasionally, but never quite herself. Physical examination disclosed the fact that there were large cavities in the apex of the left lung and flatness upon percussion in other portions of the same lung. Auscultation showed the vesicular murmur absent in the apex of the left lung, and the presence of blowing, gurgling respiration with bronchical breathing in other parts. The pulse was 120. There was no appetite. I immediately began the use of creasote, giving one minim three times a day, and gradually increasing drop by drop until the amount of 13 grains was taken (January 14, 1888) at a dose. At that date the delirium had entirely disappeared, sweats greatly diminished, cough slight, and little if any expectoration. She has gained strength, flesh and appetite. She sits up four hours a day, whereas ten weeks ago she could not get out of bed. At the date of present writing, May 1, she is around the house, gaining flesh and feeling well. The lungs have improved, and the gurgling sound of air bubbling through fluid is gone. She has taken creasote constantly for four months with two interruptions of one week each. She has taken as high as 18 grains at a dose without visible inconvenience. During this time she has had no other medication, and her diet has been principally composed of crackers and potatoes. I should state, however, that she had small quantities of whisky part of the time.—*Dr. F. L. Ladue, in Medical and Surgical Reporter.*

**Treatment of the Umbilical Cord.**—Dr. Ph. Ph. Fagonski publishes in the *Vrach* some observations on the different methods of dressing the umbilical cord after it had been tied. He employed four different methods in a hundred cases each: in the first series gypsum, in the second talc, in the third Runge's mixture (salicylic or boracic acid with potato starch), and in the fourth hygroscopic cotton-wool. In the first series erythema and intertrigo occurred five times, ulceration around the umbilicus four times, slight hemorrhage from the cord seven times, and slight suppuration twice, dry gangrene or mumification of the cord occuring in every case. In the second series ulceration occurred five times, slight hemorrhage ten times, suppuration forty-eight times, and dry gangrene seventy times. In the third series erythema and intertrigo were noted three times, ulceration twice, hemorrhage eight times, suppuration fifty-one times, moist gangrene sixty-five times, and dry gangrene thirty-five times. In the fourth series erythema and intertrigo occurred twice, ulceration three times, hemorrhage four times, omphalitis followed by death twice, suppuration twenty-nine times, moist gangrene twenty-eight times, and dry gangrene seventy-two times. With regard to the time of falling off of the cord: in the first series it was usually on the fifth day (never later) in nineteen cases on the fourth, and in four cases on the third day; in the second series separation occurred in the majority cases later than the sixth day, in six cases only being on the sixth day, in four cases on the fifth, and in one case on the fourth; in the third series the cord fell in four cases on the fifth day, and in the remaining ninety-six cases after the sixth; in the fourth series it fell on the fourth day in one case, and after the sixth in the remaining ninety-nine. It will thus be seen that the safest and best of these dressings is gypsum, but it must not be applied two liberally, for it to set up erythema. Dr. Fagonski recommends that all cases should be dressed simply with 10 grains of gypsum on cotton wool.—*London Lancet*, July 14, 1888.

**Dyspepsia.**—A lady of 56 years, long troubled with acidity of the stomach, from which she could obtain no relief, found it in the following simple combination:

℞. Bismuth subcarb............................ʒ j,
Rhei pul.................................gr. xv,
Ferri sub. carb..........................gr. xv,
Sod. bicarb..............................gr. xxx.

Mix et div. in chart No. x. Sig.—One just before meals.

In all forms of dyspepsia we should always give a constitutional remedy with one or more symptomatic medicines. Any alkali will neutralize acidity of the stomach temporarily, but there is a derangement of the secretions back of that—a vice of the organ, or may be of the constitution that needs remedying.

In the above recipe the bismuth and iron are the constitutional remedies, the rhei and soda the symptomatic. Nitrate of silver, strychnine or nux vomica are good constitutional remedies often in gastric affections. A symptom is one thing, to correct the cause of that symptom is quite frequently overlooked.

The foregoing prescription was given to a young girl of sixteen years, who had vomited every meal for nearly a year, yet she had not lost hear health, nor much flesh or strength, showing it was gastric, nervous irritability. She did not vomit a single meal after the first dose. This recipe will often allay the vomiting of pregnancy—taken half an hour before rising, followed by a bit of dry toast.—*The Medical Waif.*

**Sulphonal.**—An account of this new hypnotic has already appeared in the *Journal.* Two articles on the subject appear in a recent number of the *Berliner Med. Wochenschr*, June 18th, one by Dr. H. Rosin, of Breslau, the other by Dr. C. Oestreicher, of Berlin. Dr. Rosin tried sulphonal on eighty-two patients, besides others. In doses of 2 grammes (half a drachm) it was found almost invarialbly to have a decided hypnotic effect, without any disturbing symptoms, even when cardiac derangement was present. Such a dose Dr. Rosin considers equal to 1 6 or 1-7 of a grain of morphine, but the latter was found more efficient when th insomnia was due to cough or pain. A dose of 4 grammes (one drachm) produced a sleep lasting three or four hours in the daytime, and much longer at night; but the sleep after the dose always left a feeling of heaviness behind it. Dr. Rosin, who sticks to his text, concludes thus. "On the whole, sulphonal in doses of 2 grammes is as certain in its effects as morphine or chloral, and in cases of simple insomnia may be recommended in doses of double that strength, on account of its freedom from after-effects." Dr. Oestreicher observed the effects of sulphonal on fifty patients with nervous diseases, besides some who were phthisical, and concludes that in moderate doses—that is, 2 grammes—this drug is a non-injurious hypnotic. Respiration, pulse, and kidney-secretion were unaffected; the effects of persistent use are, of course, unknown at present It is best given in capsules or tabloids, from its insolubility in water. Oestreicher finds it without smell or taste; Rosin states that it has a slight bitter taste. Sleep sets in more slowly than after chloral or morphine in corresponding doses, but lasts longer. The editor of the *Berliner Med. Wochenschr.* confirms the above from observations of his own.—*British Med. Journal.*

**Veratrum in Pneumonia of Children.**—I had occasion to test the worth of veratrum in a case of pneumonia recently in a girl, age eleven years, with an acute at ack of the right lung. She was then very restless, with a hot, dry skin, cheeks flushed, pulse full but frequent, temperature high, respiration hurried and accompanied with the usual characteristic nervous cough. I directed a blister to the side over seat of pain, and gave a dose of calomel. I then began at once with the veratum, not a 7-drop dose, as the doctor suggested, but 5-6 of a drop, giving it myself the first day every half-hour, as I thought best, in a teaspoonful of cold water, until I had administered 8 drops of the tincture of veratrum. The admirable effects of the drug in this case were plainly seen in calming the patient, in exciting the skin to a gentle perspiration, and lowering the temperature. Suffice it to say, the veratrum was continued from my first day's visit up to the eleventh day of illness in 5-6 of a drop dose every hour, whenever the febrile movement was heightened or highly flushed cheeks were noted.

On the twelvth day the stage of resolution set in, with a steady improvement thence on with my patient. In this case I used no quinine, but gave veratrum the preference, with the view of testing its merits.—*Medical Analetic.*

**Intubation.**—The author gave a succinct review of the procedure from its accidental discovery in 1801 by Desault down to its revival in 1880 by O'Dwyer, with a synopsis of the results since achieved by the latter and others. In this review the author's own experience does good service.

A critical comparison of the relative merits and demerits of intubation and tracheotomy make a fine showing in favor of the former, so far as facility in performance and the subsequent comfort of the patient are concered. A comparison of statistics as to the final issue makes a far less satisfactory showing. In more than 20,000 tracheotomies for all forms of laryngeal occlusions, the successes were 26.5 per cent; for croup, 26.4 per cent. In 1,072 cases (in the United States) wherein intubation had been done, the successes were 26.77 per cent. It will be seen that the latter has not been in vogue a sufficient time for fair statistical comparison with the former. Time must alone fix the relative value of the two methods as life-saving measures in all forms of laryngeal occlusion. If we fail in introducing the tube, we may at once do tracheotomy. The author's successes were unequal. In the winter of 1886–87 he had fifteen cases with but one successful result. In the winter of 1887–88 he had nine cases with four successes. Two of these were in one family, and three of the successes were in succession. The type of disease is a great factor in determining success or failure

The subject of laryngeal extirpation for malignant disease was reviewed *in extenso.* The operation is one of the most formidable in surgery, results showing it to be but a desperate measure at best. It has been done one hundred and seventeen times. Of this number only nine persons are certainly recorded to have been living twelve months after operation. Of one hundred and three cases in which the operation was done for cancer, at least forty may be said to have died from the immediate effects of the operation, within a period varying from a few hours (collapse) to eight weeks.

While many surgeons consider total ablation no longer justifiable, partial extirpation would seem to give promise of much success. The comparative mortality, according to Hahn, is for total extirpation 13.7 per cent.—*Dr. Wm. Cheatham, in American Practitioner and News.*

**The Treatment of Migraine with Indian Hemp.**—For the successful exhibition of the drug, the following considerations and rules must be borne in mind: 1. Treatment by cannabis indica is not merely a palliative during one paroxysm, like the use of guarana, caffeine, morphine, nitrite of amyl, or antipyrin, and its congeners; but it is often curative, and nearly always gives some lasting relief. 2. It is necessary to persevere with the treatment for at least many weeks. Seguin obtains a promise from his patients that they will take the medicine for three months. It is usual, however, for improvement to show itself in a shorter space of time. 3. A fresh alcoholic extract only should be used; no other can be trusted for strength and efficacy. 4. One third of a grain, in pill, taken every night, or every night and morning, is sufficient to begin with; in severe cases the dose may be quickly raised to ½ grain or ⅔. More than 1 grain should not be given except to patients habituated to its use, and this amount even then should not be prescribed unless the extract to be dispensed be of the same specimen from which the patient's last prescription was made up. If this precaution be taken, there need be no fear of a toxic effect. 5. Unlike opium, no craving for further doses follows its medicinal use, and apparently it can be given up without suffering or inconvenience at any time.

It is a question if cannabis indica is entitled to a place among the neurotic poisons. Several of the authoratative works in toxicology make no mention of it. The symptoms of nervous exhilaration, expansive delirium, catalepsy, etc., which sometimes follow upon an overdose of the drug, are always alarming to the friends and family of the patient; but no case of fatal poisoning has so far been reported. Cannabis indica would seem to be more useful in women than in men, because migraine in the former is often accompanied by some irregularity in the uterine functions, and the drug has long been credited with specific action on the uterus.—*Practitioner and News.*

**Antipyrin and Spirit of Nitrous Ether.**—Eccles (*Pharmaceutical Record*) again calls the attention of druggists and physicians to the incompatibility existing between antipyrin and spirit of nitrous ether, and the great danger to life from their combination in a prescription. Because of this dangerous incompatibility not being generally known, and their having similar febrifuge properties, they are occasionally prescribed together. At least one person—a child—is known to have lost its life through this combination. Antipyrin possesses basic properties and forms salts with many acids, among which are nitrous and acetic, both of which acids either exist in the spirit of nitrous ether of the shops, or are produced in neutral solution through the action of the water used as a vehicle in the receipe. The union of antipyrin and nitrous acid forms a crystalline, greenish-colored substance, called isonitroso antipyrin, a very poisonous compound.—*Polyclinic.*

**The Treatment of Gall-Stones.**—Rosenberg, at a recent meeting of the Berlin Medical Society, stated that upon an American suggestion he had treated with large quantities of olive oil a patient, who, for five years had suffered with attacks of nephritic colic, and in vain used the most various remedies. The patient took on several evenings, without marked discomfort, from 3 to 5¼ ounces of olive oil, followed by some Cognac, and after the first dose passed three calculi, after the second, 243. In all, 26¼ ounces of olive oil were taken, in five doses, and 629 calculi counted in the stools; subsequently to which the gall-bladder, which had previously extenced markedly beyond the margin of the liver, could be no longer palpated. According to the views of American physicians, the usefulness of the olive oil depends upon its entrance into the gall-bladder, and softening the calculi.—*Wien. medizin. Presse.*

**Use of Chloroform in Tracheotomy of Children Attacked with Croup.**—M. E. Beaupere, (*Bulletin Medicale*) basing his opinion upon twenty-six observations, made for the most part in the service of M. Levrat, at the Charity, concluds as follows: Chloroform is indicated for the performance of tracheotomy in children attacked with croup. It acts in small doses and rapidly, without provoking in most cases the period of excitation. It does not increase asphyxia, it stops laryngeal spasm, and renders the operation more easy by making the child immovable and by the absence of congestion of the viens of the neck. Far from predisposing to syncope, it can, on the contrary, prevent it by diminishing inhibitory susceptibility of the region. Its use does not influence the subsequent evolution of the diphtheria. It enables one to dispense with several assistants. The only contra-indications to its employment are advanced asphyxia or lesions of the lungs.—*Indiana Medical Journal.*

**The Contagiousness of Cancer.**—Facts have already been presented which tend to prove that cancer is contagious. So far the evidence is presumptive, but it is quite probable that in the near future it will be changed to certainty.

Dr. Budd writes the *Lancet* that a patient of his who had epithelioma of the lip and refused operation, owned a terrier dog that was in the habit of licking his face. The dog contracted the cancer of the tongue and died before his master. Dr. Clemon has seen in the Royal Hospital at Liverpool a patient with cancer of the penis and testicles, which was most likely contracted by sexual intercourse with his wife who suffered from cancer of cervix uteri. A case is reported of a lady having cancer of the neck of the womb and vagina, who was nursed by a robust servant, who washed all the soiled linen. Six months after the death of the mistress, the servant was received into a hospital with cancer of the axilla of which she died —*Weekly Medical Journal.*

**Sextuple.**—Not very long ago an event occurred at Switzerland, which is without parallel in authentic history. Madame Rezzonico, wife of the Syndic of the above place, gave birth to six children, four boys and two girls. The entire half-dozen were born living, but died in a very short time. The mother is thirty-eight years of age and has had several multiple births, the children being still living. The authenticity of the above occurrence is vouched for by a number of physicians from Milan, Como and other adjacent towns who visited the woman in order to obtain the most exact information concerning this unique obstetrical event.—*Gaillard's Journal.*

**Tyrotoxicon, and its Relations to Diarrhœa in Children.**—In a recent paper concerning the nature and treatment of cholera infantum, the author has demonstrated the essentially poisonous nature of the substance which he has isolated from stale milk, and called *tyrotoxicon*. He observed that a number of children and adults, by drinking warm milk a number of hours after it had been drawn, showing evidences of poisoning. The time during which the milk had stood was sufficient for the action of an organized ferment to produce decomposition. The tryotoxicon is chemically a diagobenzol, which is decomposed at a temperature just below the boiling-point. It is formed rapidly when kept in a warm place and in a narrow covered vessel. A grain to a grain and a half given to kittens produced vio ent vomiting, diarrhœa, and death in a short time. Smaller quantities administered daily caused diarrhœa, vomiting, and rapid emaciation. The author does not maintain that this is the only poison which is obtainable from milk, and consequently from ice-cream, etc., but it is certainly one of the products of putrefaction, which has a decidedly cathartic effect. His conclusions with reference to the precautions which should be observed both in keeping milk and in preparing it for use are most important, and are as follows:

1. The cows must be healthy, and their milk must not be mixed with that from diseased cows.

2. Cows should not be fed upon the refuse from breweries or sugar-refineries nor upon other fermentescible foods.

3. They should not be allowed to drink stagnant water.

4. They should not be allowed to get overheated or worried before being milked.

5. The pastures should be kept free from poisonous plants, and the stalls and sheds kept clean.

6. If the udders are dirty, they should be carefully washed before the milking.

7. The milk should be thoroughly cooled. The vessel containing it should be kept under a stream of running water or in iced water. The milk should be kept at a temperature of not more than 60° F.

8. At no season of the year should milk be kept very long, and none should be bought which has a higher temperature than 65° F.

9. When the milk is bought, it must be evident to the buyer that it is fresh and has been properly covered. There should be no communication between the room in which the milk is brought to cool and the stable in which it is produced.

10. The milk should be kept in vessels of tin or porcelain, which should be placed in boiling water after they have been used.

The particular ferment which is the cause of tyrotoxicon is either the bacillus butyricus, or one which closely resembles it. It is often developed in the stomach, and if that viscus, or the intestine, contains undigested food, the phenomena of putrefaction will result. Children frequently overcharge their stomachs with milk merely to quench their thirst. Indigestion may result from this, and then the development of tyrotoxicon may follow. It is a matter of common observation that the digestive function is not infrequently deranged after festivals and holidays. In the treatment of diarrhœa from tyrotoxicon, the further use of milk must be suspended for the time, to check as far as possible, the multiplication of the germs. These germs are developed only in an acid medium, which indicates the use of antacid substances. The fact the alvine evacuations may be alkaline does not prove that fermentation has not taken place, for the alkalinity may be caused by the alkaline serum of the blood which has been effused into the intestines. The value of subnitrate of bismuth in such cases is due to its germicidal properties, and the same is true of the salicylate of soda and calomel. The latter must be given, however, in small doses, and will usually be found very effective.—*Arch. of Pediatrics.*

**Cytisin in Migraine.**—Dr. Kraepelin has found cytisin, the active principle of laburnum, and other species of cytisus, very valuable in some forms of migraine which appear to be due to a dilated condition of the vessels, this powerful and highly toxic substance acting as a vaso-constrictor. In the case of a young woman of hysterical tendencies, who had very violent attacks of migraine, associated with vomiting, sleeplessness, photophobia, swimming of the eyes, and unbearable hemicrania, where all ordinary remedies had proved useless, the hypodermic injection of 0.003 gramme of nitrate of cytisin had an immediate effect. In subsequent attacks it also succeeded, except once when an attempt was made to administer it by the mouth, when it was immediately vomited. The dose was increased ultimately to 0.005 gramme. Sleep could frequently, but not always, be induced by paraldehyde, in 2-drachm doses. Dr. Kraepelin found that cytisin had a negative, sometimes even an injurious effect, in cases where the migraine was accompanied by a good deal of spasmodic action, so that it is only to be recommended in migraine of a paralytic type.—*Lancet.*

**Goitre Can be Cured.**—Dr. W. B. Cauble (*Medical Brief*, June, 1888,) says: I have witnessed the cure of many of these enlarged thyroids while at college, and that has only been a short time since. I will not say definitely, whether or not the treatment is a specific, but if the case is seen early, I do not hesitate to say that it can be cured if medicine is used regularly and constantly. The treatment may have to be continued for a period of from two to six months or longer.

It consists in the injection of a five per cent. solution of carbolic acid into the goitre, using from 7 to 15 minims at each sitting, this to be repeated every five days, and if the growth is large it may be injected on both sides at one sitting, so as to check its progress much earlier. In connection with the above the patient should receive 3 drops of Donovan's solution of arsenic three times daily.

After the injection the patient may feel somewhat dizzy, but this sensation will soon pass off. Care should be taken while injecting, not to penetrate a vessel.

**The Relative Value of the Bromides.**—Dr. Stewart, in *Polyclinic*, says of the relative value of the bromides: Cory (*Brit. Med. Journal*), believing that the basic element is the cause of the unpleasant effects produced when potassium bromide and iodide are given in large and continuous doses, has, in private and hospital practice, for several years prescribed exclusively the sodium instead of the potassium salts with marked advantages as regards the unpleasant symptoms. and with no corresponding diminution in the beneficial effects of these drugs. Apart from the former being less hurtful, they should be more efficient in the same dose, for, from the combining weight of sodium being 22 99 and potassium 39.04, every 10 grains of sodium bromide contain 7.76 grains of bromine, and every 10 grains of potassium bromide contain 6.72 grains of bromine; so that in order to prescribe the same weight of bromine, we must give instead of 10 grains of bromide of potassium only 8.6 grains of bromide of sodium. So also with the iodide of potassium, a 10-grain dose is represented by a 9-grain dose of iodide of sodium.

**Oil of Corn.**—Prof. Curtman, of St. Louis, thus describes this new product: "Oil from embryo of Indian corn, in unrefined state, has a specific gravity of 0.916 at 15°C., which is nearly that of pure olive oil (0.915 to 0.918.) The elaidin test shows the presence of a large quantity of olein, intermediate in quantity between olive and cotton seed oils. Its color is a pale yellow brown; its odor and taste that of freshly ground corn meal. It belongs to the non-drying group of the vegetable oils, experiments showing that a very thin layer on paper does not in three weeks' time form a pellicle on the surface exposed to air. In this respect it closely resembles the oils of olive, almond, colza, rape-seed, etc. It does not very rapidly become rancid by exposure to air, and in this regard compares favorably with the best oils. Its use produces no specific purgative effect any more than olive oil. With ammonia or solutions of caustic alkalies it rapidly saponifies, forming a white soap."—*American Journal of Pharmacy.*

**Action of Boiling Water on Typhoid Baccilli.**—Wilchur, of St. Petersburgh, has found that when a volume of boiling water equal to that of gelatine culture of typhoid bacilli is used on the culture the bacilli are only partly destroyed, and that when the volume of water is double that of the culture all the bacilli are killed. Experiments on the dejecta of typhoid patients showed that when four times the volume of water was added to the dejecta, the bacilli were invarably destroyed. It seems then, that this is an easy and certain method of disinfecting typhoid stools. —*Journal of the American Medical Association.*

**A Cure for Drunkeness.**—A half ounce of ground quassia steeped in a pint of vinegar, is recommended highly as a cure for drunkenness. A teaspoonful in a little water should be taken every time the liquor taste is felt. It satisfies the cravings and produces a feeling of stimulation and strength.—*Medical World.*

